# Utility of Serial Repetitive Nerve Stimulation in Differentiating Pharmacological From Immunological Exacerbation of Myasthenia Gravis: A Case Report

**DOI:** 10.7759/cureus.94916

**Published:** 2025-10-19

**Authors:** Akira Hanazono, Keita Yasuda, Yoshiko Takahashi, Yui Sanpei

**Affiliations:** 1 Neurology, Akita University Graduate School of Medicine, Akita, JPN

**Keywords:** blood level monitoring, chronic kidney disease, cibenzoline, elderly patients, waning

## Abstract

Myasthenia gravis (MG) can be exacerbated by certain medications. However, the contribution of many suspected medications remains uncertain, often based on case reports and confounded by variable drug responses and the fluctuating nature of MG. Objective drug evaluations are needed to establish causation. This report examines cibenzoline, an antiarrhythmic suspected of exacerbating MG, and proposes a method for assessing the causal role of medications in MG exacerbations through daily repetitive nerve stimulation (RNS) correlated with drug levels. We present a case of a 78-year-old man with stable ocular MG, treated with prednisolone and cibenzoline, who developed a myasthenic crisis requiring intubation. The patient’s worsening chronic kidney disease raised concerns about potentially increased cibenzoline levels and their role in the crisis. Serial co-evaluation of RNS studies and cibenzoline blood levels over three consecutive days demonstrated a strong correlation between cibenzoline levels and the severity of neuromuscular junction fatigue. Rapid clinical and electrophysiological recovery within a day following withdrawal, and subtle improvement after the drop in blood cibenzoline level, suggested a primary pharmacological mechanism rather than an immunological one, as immunological improvement usually requires at least several days. This case highlights the importance of serial electrophysiological assessments combined with drug-level monitoring to evaluate the causality in suspected medication-induced MG exacerbations. Given the increasing number of elderly patients with MG or chronic kidney disease and the potential for undiagnosed MG, the risk of iatrogenic adverse events could increase. The proposed methodology, which utilizes serial RNS with blood concentration monitoring of the suspected drug, can facilitate an accurate evaluation of the causal relationship in pharmacological versus immunological exacerbations of MG.

## Introduction

Myasthenia gravis (MG) can be exacerbated by various drugs that potentially disrupt neuromuscular junctions (NMJs) [1,2]. The rate of drug-induced exacerbation of MG is 19% [3]. However, the contribution of many suspected drugs remains uncertain [1], often based on case reports [4], and confounded by inter-patient variability in drug response and the fluctuating nature of MG symptoms. Furthermore, neurological evaluation can be challenging in intubated and sedated patients experiencing myasthenic crisis. Consequently, distinguishing between pharmacological and immunological factors contributing to myasthenic fluctuations is difficult when drug-induced NMJ dysfunction is suspected. Given these complexities, a simple observation of myasthenic worsening following drug administration is insufficient to evaluate the causality. As a result, many drugs remain “suspected” or “anecdotal” MG exacerbators, which can complicate clinical decision-making because these drugs often provide benefits for other coexisting conditions [4].

The exacerbation of MG by cibenzoline has been suggested by several case reports, especially in patients with drug accumulation with chronic kidney disease (CKD) [5-10]. The plasma elimination half-life was prolonged, ranging from 7.4 to 23.6 hours, due to impaired renal function [11]. The reported safe blood concentration of cibenzoline is inconsistent in the literature. While some cardiology studies indicate a safe and effective range of 300 to 1500 ng/mL [12], others define the therapeutic range as 300 to 1000 ng/mL [7]. However, its contribution remains anecdotal, and the impact of antiarrhythmics in general on NMJ is undetermined [1,4].

Here, we present a case of myasthenic crisis in which serial evaluations of electrophysiological studies and blood cibenzoline levels helped differentiate a pharmacological cause from an immunological one. This report aims to demonstrate the clinical utility of this systematic approach in evaluating suspected drug-induced MG exacerbations.

## Case presentation

A 78-year-old man with a four-year history of stable ocular MG presented to our hospital. He had positive acetylcholine receptor (AChR) antibodies (1.8 nmol/L) but no thymoma, suggesting a low likelihood of progression to generalized MG. His MG was well managed only with oral prednisolone 5 mg/day. He had been taking cibenzoline for several years to prevent supraventricular cardiac arrhythmia without prior adverse effects. His medical history also included worsening CKD. Over a month, he experienced a gradual worsening of fatigue and ocular MG symptoms. There were no identifiable common triggers for myasthenic exacerbation, such as contraindicated medication, surgery, trauma, infection, or emotional stress. His symptoms progressed to include masseter fatigue, dysarthria, dysphagia, sputum retention, and finally aspiration pneumonia. Prior to admission, dehydration and antibiotic treatment (sulbactam/ampicillin) at another hospital worsened his CKD, with a glomerular filtration rate from 29.1 mL/minute to 16.1 mL/minute (by the Cockcroft Gault equation) before the hospitalization. Upon admission, he was treated with intravenous immunoglobulin for five days and a steroid pulse (methylprednisolone 1000 mg/day for three days). Noninvasive positive pressure ventilation (NIPPV) was initiated. However, on the third day of these immunological treatments, increased sputum production led to NIPPV failure, and blood gas analysis revealed PCO2 of 77 mmHg, necessitating intubation and mechanical ventilation. Table 1 shows laboratory data at the worsening.

**Table 1:** Laboratory data at the worsening of myasthenia gravis ALT - alanine aminotransferase; AST - aspartate aminotransferase; BUN - blood urea nitrogen; Cl - chloride; CRE - creatinine; CRP - C-reactive protein; HGB - hemoglobin; K - potassium; Na - sodium; PLT - platelet; WBC - white blood cell

	Value	Unit	Reference Range
WBC	7000	/μL	3300-8600
Neutrophil	91.0	%	38.5-80.5
HGB	15.3	g/dL	13.7-16.8
PLT	19.7	×104/μL	15.8-34.8
CRP	0.30	mg/dL	0-0.14
BUN	56.9	mg/dL	8-20
CRE	3.02	mg/dL	0.65-1.07
Na	135	mEq/L	138-145
K	4.7	mEq/L	3.6-4.8
Cl	101	mEq/L	101-108
AST	29	U/L	13-30
ALT	24	U/L	10-42

**Figure 1:**
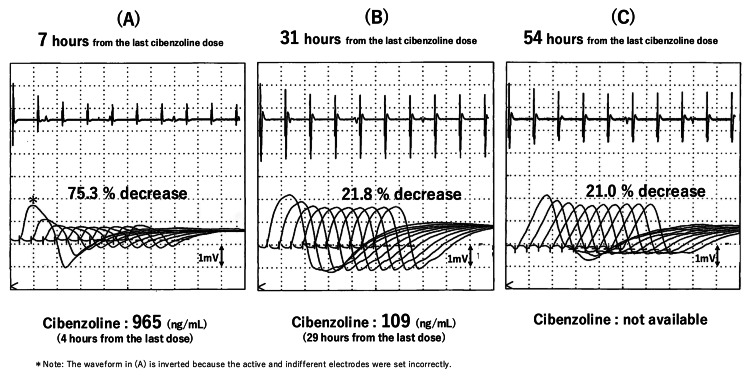
Repetitive nerve stimulations after cibenzoline withdrawal A 3 Hz repetitive nerve stimulation of the trapezius muscle, using the left accessory nerve, shows severe neuromuscular junction (NMJ) fatigue with a 75.3 % J-shaped decrement seven hours from the last cibenzoline dose. Concurrently, the cibenzoline blood level is high at 965 ng/mL (A). Thirty-one hours from the last dose, the decrement has drastically improved to 21.8%, along with a decreased blood level of 109 ng/mL (B). Fifty-four hours from the last dose, the change in decrement rate becomes subtle at 21.0 %, suggesting that the electrophysiological change from A to B appears to derive from pharmacological NMJ fatigue rather than immunological one (C).

The patient was unable to touch his face or lift his knees. This lack of response to acute immunotherapy by day 3 and the worsening CKD prompted consideration of cibenzoline's potential contribution. A blood cibenzoline concentration measured four hours after the last dose was moderately high at 965 ng/mL. Concurrently, 3 Hz repetitive nerve stimulation (RNS) to the left accessory nerve demonstrated severe NMJ fatigue of the trapezius muscle with a 75.3% decrement (Figure 1A). The day following cibenzoline discontinuation, his MG symptoms nearly disappeared, and RNS improved to a 21.8% decrement (Figure 1B). The cibenzoline blood concentration simultaneously decreased to 109 ng/mL. The rapid clinical improvement within a day after cibenzoline withdrawal suggested a primary pharmacological effect, rather than an immunological response. Furthermore, the subsequent electrophysiological improvement was subtle (Figures 1B-1C), and MG symptoms remained almost asymptomatic. These observations supported the assessment that pharmacological NMJ dysfunction was the dominant etiology. Three days from the last cibenzoline dose, the patient was successfully weaned from mechanical ventilation. Cibenzoline was replaced with verapamil, and no significant arrhythmias were observed.

## Discussion

This case report suggested the utility of serial RNS in differentiating pharmacological from immunological exacerbation of MG. We propose that this methodology, along with blood level monitoring, should be considered for other suspected medications during a myasthenic crisis. Even if drug level testing is unavailable, serial daily electrophysiological evaluations after drug withdrawal can reveal drug contributions because immunological therapies generally require several days to weeks to become effective [13].

RNS at 3 Hz is a standard technique for evaluating NMJ fatigability, a method applicable not only to MG but also to drug-induced neuromuscular blockade [14,15]. However, it is noteworthy that immunological worsening of MG shows different electrophysiological decrement rates with individual differences, and the single-point RNS study could not be correlated with the severity [14]. In contrast, the severity of pharmacological NMJ disturbances is correlated with decrement rate, and rapid improvement of RNS usually indicates improvement of NMJ functions, which is based on the anesthesiologic studies [15]. Therefore, as shown in our case, at least three consecutive days of RNS might show initial drastic improvement followed by subtle amelioration; this rapid RNS improvement could distinguish the pharmacological from immunological NMJ fatigue.

Elderly patients are at increased risk of iatrogenic adverse events during MG treatment [16] and exhibit age-related changes in pharmacokinetics [17]. In elderly patients with prolonged ocular MG without thymoma, as in the case with a four-year history of stable ocular MG, progression to generalized MG is uncommon [18,19]. Therefore, iatrogenic factors should be considered in similar cases to the present case.

The MG worsening with the cibenzolin accumulation is likely due to its anticholinergic effects [9]. Even in non-MG patients, extreme over-accumulation of cibenzoline can induce myasthenic symptoms with respiratory failure [5,6,8,10]. However, reported safe blood levels vary [6,8-10,12], and are primarily based on studies in non-MG patients. In cardiology, “safe” cibenzoline levels up to 1500 ng/mL have been reported [12], significantly higher than the 965 ng/mL observed in our patient during a myasthenic crisis. Clinicians should be aware that even normal or moderate accumulation of cibenzoline can trigger a myasthenic crisis, particularly given the potential for underdiagnosis of MG in elderly patients [20] and the increasing number of patients with CKD, which could increase the risk of unexpected iatrogenic deterioration.

As a limitation, baseline data on the patient's renal function and drug levels prior to the exacerbation were unavailable. Furthermore, strictly simultaneous measurements of serum cibenzoline concentration and RNS decrement were lacking, with a maximum interval of three hours between the corresponding measurement points. Furthermore, we acknowledge that infections are the most common contributor to MG worsening, accounting for 30% of cases [3]; thus, aspiration pneumonia was an undeniable precipitating factor for the crisis. It is also important to consider that steroid-related initial worsening might have contributed to the overall clinical decline. However, these are common challenges in acute clinical practice, and our method, focusing on the rapid change in blood level monitoring and RNS studies correlated with clinical improvement, could help distinguish in such real-world scenarios. On the other hand, the rapid improvement observed in this case is likely influenced by the relatively short half-life of cibenzoline. For medications with longer washout periods or other mechanisms for MG worsening, the correlation between withdrawal and RNS improvement may be less immediate, potentially requiring a more extended observation window.

Therefore, the rapidity of the improvement of myasthenia and RNS studies, along with the quick decrement of blood concentration of the suspected drugs, should be evaluated in future studies.

## Conclusions

Serial co-evaluations of RNS studies with blood levels over three consecutive days helped distinguish the pharmacological worsening of NMJ function from the immunological one. This methodology should be considered for other MG patients experiencing worsening symptoms while taking suspected medications, particularly given the increasing number of elderly patients with MG and CKD, who are at higher risk for iatrogenic complications.
